# Co-occurrence of Meningioma and Intracranial Aneurysm: A Systematic Review

**DOI:** 10.7759/cureus.52919

**Published:** 2024-01-25

**Authors:** Fatimah H Albahrani, Jasmine A Alturaiki, Abdulaziz Y Alahmed, Jinan M Aljasem, Munif M Alshammari, Abdullah S Alali, Abdulelah Y Aldabbab, Ali A Alhelal, Abdu Alkhairy

**Affiliations:** 1 General Practice, King Faisal University, Al-Ahsa, SAU; 2 Medical School, King Faisal University, Al-Ahsa, SAU; 3 Neurosurgery, General Directorate of Health Affairs, Aseer, SAU; 4 Skull Base and Vascular Neurosurgery, General Directorate of Health Affairs, Aseer, SAU

**Keywords:** meningioma, coexistence, cerebral aneurysm, rupture of intracranial aneurysms, subarachnoid hemorrhage, intracranial aneurysm

## Abstract

A complete understanding of the rare neurosurgical phenomenon of co-occurring meningioma and intracranial aneurysm is important to improve the quality of life and decrease future complications in these patients. In this review, we searched the literature for cases of this rare phenomenon to highlight the most important historical, investigation, and treatment-related factors to improve the accuracy of intraoperative procedural decisions. We searched the PubMed database for case reports on this neurological rare phenomenon to create organized data for our review. Then, we extracted information from these cases and organized it in a table. We identified 19 cases in the literature. In the published studies, there was a predominance of the female sex (73.68%). The mean age of the patients was 54.11 years, with the cases relatively evenly distributed among patients in their 30s, 40s, 50s, 60s, and 70s. Posterior communicating artery aneurysm was the most common among the 19 cases. For meningioma, the frontal lobe and clinoid were the two most affected locations, and the meningothelial histopathology was the most common. Complete tumor resection and aneurysmal clipping were done for the majority of the cases (57.8%) unless there was a complication that deferred simultaneous intervention. Fortunately, most patients (78.95%) recovered completely after surgery. The coexistence of meningioma and intracranial aneurysm has a very high cure rate, postoperative symptom resolution, and a very low recurrence rate. For most cases, neuroimaging investigations are recommended for simultaneous management. This imaging can also highlight other potentially suspicious findings.

## Introduction and background

Subarachnoid hemorrhage (SAH) and other potentially catastrophic problems can result from the rupture of intracranial aneurysms, a cerebrovascular condition characterized by aberrant bulging of the cerebral artery [1]. There are three types of intracranial aneurysm, namely, saccular, which represents 90% of all cases, fusiform, and dissecting [2]. The circle of Willis is the most commonly affected arterial structure due to its high wall shear stress, which might restrict blood flow and cause an intracranial aneurysm [3-5]. The prevalence of intracranial aneurysms ranges from 2% to 5% in adults, and the rupture rate ranges from 8 to 10 per 100,000 each year [3]. Meningioma frequently develops from the meninges, specifically the arachnid layer, that surrounds the brain and spinal cord in the form of a benign central nervous system tumor [6]. The World Health Organization (WHO) classifies meningiomas into three grades, namely, grade I, benign; grade II, atypical; and grade III, malignant [7]. Atypical meningiomas are characterized by more tissue and cell abnormalities, and they expand faster and recur more often than benign tumors [7]. Unfortunately, grade III malignant meningiomas have the worst prognosis. Their growth and invasion rates are the highest of the three grades [7]. A large proportion of primary brain tumors (37.6%) are meningiomas, which also account for nearly 50% of all benign brain tumors, with a higher incidence in adults [6,8]. Interestingly, the co-occurrence of intracranial aneurysm and meningioma has been reported in several studies. The debate about the accurate pathophysiological process is mainly due to the low epidemiological prevalence of this co-occurrence: seven of 956 (0.73%) [9], two of 1,500 cases (0.13%) [10], and five of 426 cases (1.17%) [11]. Moreover, Handa et al. [9] reported that of 134 tumors with aneurysms, 40 were meningiomas (29.85%) [9]. These facts prompt the need to study and discuss the factors that influence the co-occurrence of meningioma and intracranial aneurysms. Hence, in this review, we identified published case reports of this phenomenon.

The main aim of this study is to evaluate the documented cases reporting the co-occurrence of meningioma and intracranial aneurysms published between 1981 and 2023 and specifically discuss three points of view (preoperative, intraoperative, and postoperative considerations) to identify the factors that influence meningioma and intracranial aneurysms coexistence, leading to a better understanding of these rare cases and improving the intraoperative procedural decisions. Second, this study aims to examine the potential correlation and underlying mechanisms of the co-occurrence of intracranial aneurysm and meningioma. Gathering information regarding this neurosurgical phenomenon can lead to improvement in the quality of life of these patients and minimize the risk of any future complications by exploring and implementing the most effective management strategies specifically designed for such cases.

## Review

Methodology

**Figure 1:**
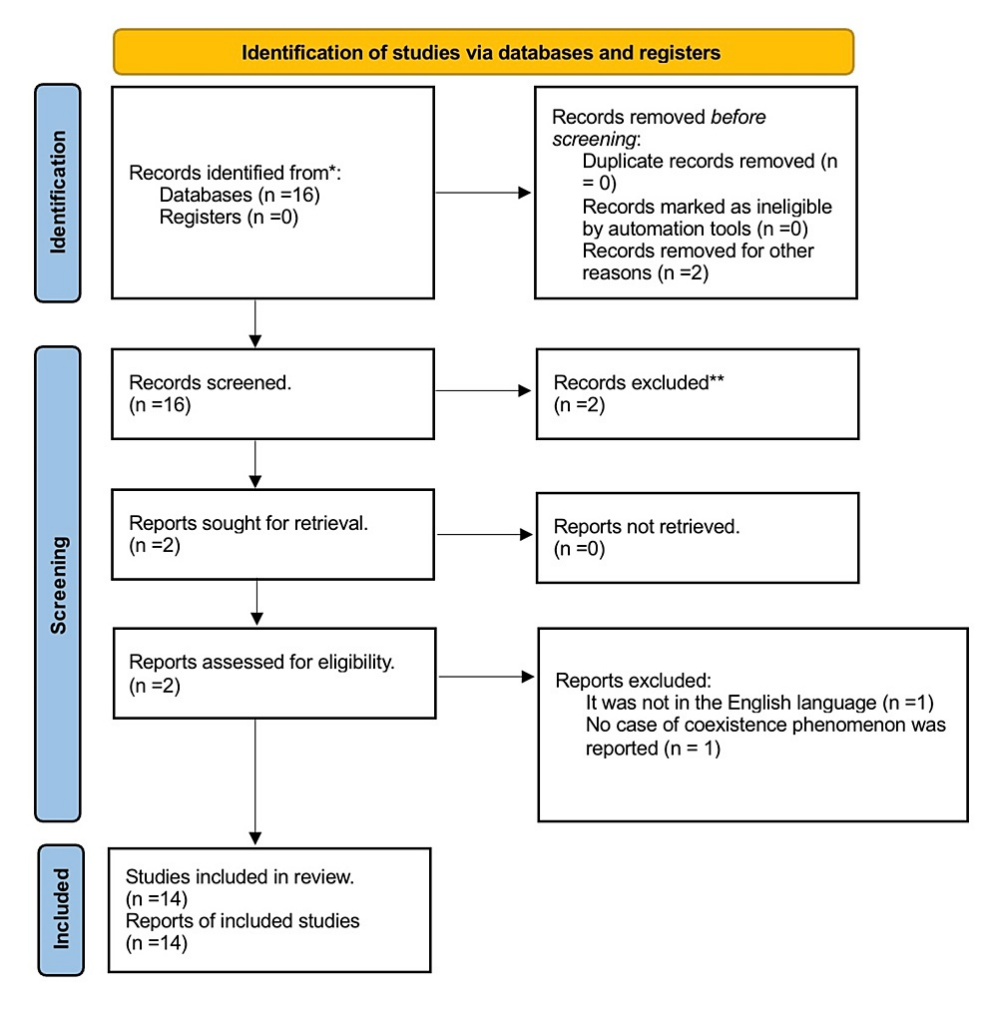
Preferred Reporting Items for Systematic Reviews and Meta-Analyses flowchart.

We performed a preliminary search of the PubMed database to find cases where meningioma and cerebral aneurysms coexisted. We included all English-language cases from the PubMed database that were reported to have both intracranial aneurysm and meningoma concurrently. We used the following terms for the advanced search to identify all the specific case reports: ((Aneurysm) AND (Meningioma) AND (Case Report) AND (Coexistence)). The time filter was from 1981 to 2023. Studies that did not appear in PubMed, were not written in English, or focused solely on meningioma or intracranial aneurysm without discussing their coexistence were omitted. In total, 14 of the 16 studies were incorporated into our final review (Figure 1). A total of 19 distinct cases were found in these 14 studies. Quality assessment of the study was done using the Critical Appraisal Skills Programme Checklist [12].

Results

Table 1 outlines the reported age, sex, comorbidities, and symptoms in the case studies. The age of the patients ranged from 34 to 71 years, with a mean of 54.11 years, a median of 55 years, and a mode of 55 years. Among the 19 patients, three were in their 30s (15.79%), four were in their 40s (21.05%), five were in their 50s (26.32%), four were in their 60s (21.05%), and three were in their 70s (15.79%). Of the 19 patients, five were male (26.32%) and 14 were female (73.68%). Hypertension was the most common comorbidity (six of 19 cases, 31.58%). Overall, one (5.26%) patient had a history of allergic reaction to contrast medium, one patient had diabetes mellitus (5.26%), one (5.26%) patient had atherosclerosis, one (5.26%) patient had ulcerative colitis, and one (5.26%) patient had restless legs syndrome and parkinsonism. Eleven authors did not mention the patients’ comorbidities. None of the authors mentioned the patients’ habits (e.g., smoking and alcohol use). The predominant presenting symptom was headache in 11 (57.89%) cases. Other prevalent symptoms include ptosis (10.53%), memory disruption (10.53%), speech disturbance (10.53%), and seizures (10.53%). Additional symptoms, each reported in 5.26% of cases, included weakness in the lower limb, unsteady gait, drowsiness, confusion, decline in performance, behavioral disturbance, diminished vision, diplopia, vertigo, vomiting, and loss of consciousness.

**Table 1:** Characteristics of individuals in the case reports.

Author	Age	sex	Comorbidities	Symptoms
Javalkar et al., 2009 [11]	70	Female	N/A	Sudden severe headache and diminished vision in the right eye
Javalkar et al., 2009 [11]	55	Female	N/A	Speech disturbance
Javalkar, et al., 2009 [11]	37	Female	N/A	Not mentioned
Javalkar et al., 2009 [11]	63	Female	N/A	The worst headache of her life
Javalkar, et al., 2009 [11]	61	Female	N/A	Sudden severe headache
Shigemori et al., 1991 [13]	49	Male	N/A	Headache, memory disturbance, and weakness in the right lower limb
Suslu et al., 2009 [14]	41	Female	N/A	Chronic headache
Paraskevopoulos et al., 2011 [15]	55	Female	Allergy to contrast medium	Two episodes of vertigo
Alnaami et al., 2013 [16]	34	Male	N/A	Difficulty sleeping, and the worst headache of his life
Waqas et al., 2015 [17]	60	Female	Hypertension	Drowsiness and inability to open her left eye for 10 days
Eulate-Beramendi et al., 2017 [18]	71	Female	Hypertension and diabetes mellitus	Gait disturbance
Eulate-Beramendi et al., 2017 [18]	67	Female	Ulcerative colitis	Confusion and behavioral disturbance
Zhou et al., 2017 [19]	53	Male	Hypertension and atherosclerosis	Sudden onset of headache and vomiting
Yip et al., 2019 [20]	71	Male	Hypertension, restless legs syndrome, and parkinsonism	Headache, unsteady gait, slurred speech, and decline in memory and performance
Papadimitriou et al., 2020 [21]	55	Female	N/A	One episode of a generalized tonic-clonic seizure
Tanaka et al., 2022 [22]	52	Female	Hypertension	Persistent morning headache
Algburi et al., 2022 [23]	48	Female	N/A	Loss of consciousness and recurrent seizures
Wei et al., 2022 [24]	38	Male	N/A	Headache
Onyia et al., 2023 [25]	48	Female	HTN	Headache with complete left ptosis and diplopia

Table 2 presents the specific characteristics of meningioma patients based on reported cases. The frontal lobe and clinoid were the most frequently affected locations (each in four of 19 cases, 21.05%). Other locations included sphenoid (three of 19 cases, 15.79%), the pterional region (two of 19 cases, 10.53%), and the falcine sinus (two of 19 cases, 10.53%). Less frequently involved regions were petroclival, sella, and intrahemispheric, each reported in one of 19 cases (5.26%). Histopathological classification was not specified in 10 of 19 cases (52.63%). Among the specified cases, eight of 19 (42.10%) were grade I meningiomas. Of these, five were meningothelial (62.5%), one fibroblastic (12.5%), one psammomatous (12.5%), and one transitional (12.5%). A single case (5.26%) was described as a grade II fibrous tumor with hemangiopericytoma. It illustrates the aneurysmal arteries that are connected to meningiomas. The internal carotid artery (ICA), middle cerebral artery (MCA), anterior cerebral artery (ACA), posterior communicating artery (PcomA), anterior communicating artery (AcomA), pericallosal artery, and frontopolar artery were identified. ICA aneurysms occurred in five of 19 cases (26.31%), with ophthalmic artery involvement in 40% of these cases and cavernous segment involvement in 20%. PcomA aneurysms were observed in six of 19 cases (31.58%), predominantly on the right side (66.67%). AcomA aneurysms were present in four of 19 cases (21.05%), with 25% on the left side. MCA aneurysms occurred in three of 19 cases (15.79%), mostly on the right side (66.67%). ACA aneurysms were identified in two of 19 cases (10.52%), evenly distributed between the left and right sides. Additionally, aneurysms of the left pericallosal artery and right frontopolar artery were observed in two of 19 cases (10.52%) and one of 19 cases (5.26%), respectively, with precise locations not reported for the remaining cases. Regarding management, a majority of patients underwent complete tumor resection and aneurysmal clipping (11 of 19 cases, 57.89%). Others were treated by complete tumor resection only (one of 19 cases, 5.26%), partial tumor resection and aneurysm embolization (one of 19 cases, 5.26%), and craniotomy resulting in SAH (one of 19 cases, 5.26%). The second most common management involved complete tumor resection and aneurysmal embolization (three of 19 cases, 15.79%). Lastly, in one of 19 cases (5.26%), the patient underwent preoperative embolization before complete tumor resection. The prognosis was generally favorable, as shown in Table 2, with most patients experiencing full recovery after surgery (15 of 19 cases, 78.95%). One (5.26%) patient reported a reduction in headaches. However, less successful outcomes were noted, with one (5.26%) patient still experiencing headaches, a second patient (5.26%) developing a hemorrhage in the left Sylvian fissure/mild right hemiparesis, and another patient (5.26%), unfortunately, died two days after surgery.

**Table 2:** Characteristics of meningioma and the affected artery in the reported cases. PcomA = posterior communicating artery; ICA = internal carotid artery; SAH = subarachnoid hemorrhage; MCA = middle cerebral artery; ACA = anterior cerebral artery

Author	Location	Histopathology	Aneurysmal artery	Management	Prognosis
Javalkar et al., 2009 [11]	Right pterional meningioma	N/A	Bilateral PcomA	Complete tumor resection/aneurysmal clipping	Full recovery after surgery
Javalkar et al., 2009 [11]	Clinoidal meningioma	N/A	Right PcomA	Complete tumor resection/aneurysmal clipping	Full recovery after surgery
Javalkar et al., 2009 [11]	Left petroclival meningioma	N/A	Incidental ICA	Complete tumor resection/aneurysmal clipping	Full recovery after surgery
Javalkar et al., 2009 [11]	Planum sphenoidale meningioma	N/A	AcomA complicated with SAH	Complete tumor resection/aneurysmal clipping	Full recovery after surgery
Javalkar et al., 2009 [11]	Incidental clinoidal meningioma	N/A	Right PcomA	Aneurysmal clipping	Full recovery after surgery
Shigemori et al., 1991 [13]	Left frontal meningioma	Typical meningotheliomatous meningioma, grade I	M1 portion of left MCA	Complete tumor resection/aneurysmal clipping	Uneven, full, still complaining of headache
Suslu et al., 2009 [14]	Left frontal meningioma	Fibroblastic meningioma, grade I	Cavernous segment of ICA	Complete tumor resection only	Full recovery after surgery
Paraskevopoulos et al., 2011 [15]	Left anterior clinoidal meningioma	N/A	Right MCA and left ACA	Complete tumor resection/aneurysmal clipping	Hemorrhage in the left Sylvian fissure/mild right hemiparesis
Alnaami et al., 2013 [16]	Intrahemispheric meningioma	Meningioma with embolization material within the tumor, grade I	The distal left pericallosal artery	Partial tumor resection/aneurysmal embolization	Full recovery after surgery
Waqas et al., 2015 [17]	Clinoidal meningioma	N/A	ICA	Complete tumor resection/aneurysmal clipping	Full recovery after surgery
Eulate-Beramendi et al., 2017 [18]	Left posterior meningioma	Meningothelial meningioma, grade I	Right PcomA aneurysm	Complete tumor resection/aneurysmal embolization	Full recovery after surgery
Eulate-Beramendi et al., 2017 [18]	Left medial sphenoidal wing meningioma	Meningothelial meningioma, grade I	Incidental right PcomA	Craniotomy led to SAH	The patient died after two days of the surgery
Zhou et al., 2017 [19]	Left medial sphenoidal wing meningioma	N/A	Left ICA-ophA	Complete tumor resection/aneurysmal embolization	Headaches were reduced
Yip et al., 2019 [20]	Right frontotemporal	Fibrous tumor/hemangiopericytoma, grade II	Bifurcation of the right MCA	Complete tumor resection/aneurysmal clipping	Full recovery after surgery
Papadimitriou et al., 2020 [21]	Falcine meningioma	Meningothelial meningioma, grade I	Left pericallosal artery, left AcomA, and right frontopolar artery	Preoperative embolization before complete tumor resection	Full recovery after surgery
Tanaka et al., 2022 [22]	Right frontal meningioma	Transitional meningioma, grade I	Left ICA-OphA	Complete tumor resection/aneurysmal embolization	Full recovery after surgery
Algburi et al., 2022 [23]	Ipsilateral frontal meningioma	N/A	AcomA	Complete tumor resection/aneurysmal clipping	Full recovery after surgery
Wei et al., 2022 [24]	Right paraflacine meningioma	Meningothelial meningioma, grade I	AcomA and A1, A2 of right ACA	Complete tumor resection/aneurysmal clipping	Full recovery after surgery
Onyia et al., 2023 [25]	Sellar meningioma	Psammamatous meningioma, grade I	Left PcomA	Complete tumor resection/aneurysmal clipping	Full recovery after surgery

Discussion

Explanation for the Co-occurrence of Meningioma and Intracranial Aneurysm

An aneurysm is a defect in the wall structure, specifically in the internal elastic lamina and media, that eventually causes a focal fragile segment in the arterial wall that expands outward [25]. Most intracranial aneurysms remain silent during the lifetime of the affected patient [25]. One hypothesis is that an aneurysm starts initially with a hemodynamic insult that leads to a complex inflammatory process involving matrix metalloproteinases (MMPs), vascular smooth muscle cells (VSMCs), macrophages, and oxidative stress [25]. In addition, oxidative stress also plays a major role in endothelial injury and results in the accumulation of free radicals due to increased production or decreased removal [25]. Furthermore, inflammation follows an endothelial injury [25]. This process involves macrophages, mast cells, T cells, cytokines, and inflammatory mediators, which modulate the phenotype of VSMCs [25]. This modulation leads to disruption of the internal elastic lamina and induces dysregulation of the internal elastic lamina alongside dysregulation of collagen synthesis and extracellular matrix [25]. The late steps in the growth of an intracranial aneurysm involve VSMC apoptosis, which results in further thinning of the media and an increased risk of rupture [25]. There are several theories as to how a meningioma and an aneurysm can co-occur [26,27]. One theory suggests that meningioma with increased blood flow might be associated with a higher risk of aneurysm [26,27]. Another theory proposes the possibility of meningioma adhesions to the arterial adventitia, which might damage the arterial wall and promote the formation of an aneurysm [26,27]. However, it remains unclear why meningioma and an aneurysm can co-occur [26,27]. Moreover, tumor size seems to have an impact on the formation of intracranial aneurysms [28]. A growing meningioma increases intracranial pressure, which, in turn, leads to a proportional increase in blood pressure to ensure a constant cerebral perfusion pressure is maintained [28]. The increase in blood pressure may augment hemodynamic stress around the meningioma [28]. This hemodynamic stress can result in remodeling and degeneration of internal elastic lamina [28]. Consistent with this expected phenomenon and the increased blood flow theory, the majority of intracranial aneurysms are associated with a wide variety of tumor types [29]. Higher percentages have been recorded for blood flow-dependent tumors, including meningioma (29.3%-44%), glioma (27.5%-38%), pituitary adenoma (11%-20.6%), lymphoma, craniopharyngioma, chordoma, epidermoid tumor, dermoid tumor, and choroid plexus adenoma [29]. Of note, based on the literature, hormones do not have a direct effect on aneurysm formation [30]. However, some studies have suggested that hormones, such as estrogen, can potentially play a role in the co-occurrence of meningioma and an intracranial aneurysm given the predominance of the female sex [31]. Thus, researchers have suspected a hormonal contribution to this pathological phenomenon [31].

Age

In the 19 cases, the age mean was 54.11 years, and more than a quarter of the patients were in their 50s [11-25]. Starting from the fifth decade of life, the elasticity of a vessel along with its ability to recoil begins to decrease significantly [32]. These changes increase the pressure upon weak foci in the arterial wall [32]. The outcome could be endothelial dysfunction with inflammation, oxidative stress, and, finally, aneurysm formation [32]. Multiple autopsy studies have revealed that the incidence of intracranial aneurysms increases with age [32]. Because age is a non-modifiable risk factor, primary prevention strategies, such as encouraging a healthy lifestyle, should be applied to reduce the burden of age and decrease the future incidence of intracranial aneurysms [32].

Sex

The majority of patients were women (14 of 19 cases, 73.68%) [11-25]. There is a female predominance for meningioma and intracranial aneurysms, and the estrogenic window is suspected to be the most reliable risk factor for these conditions [33]. Indeed, the well-being of women, including arterial health, depends on estrogen [33,34]. This hormone inhibits MMPs, an action that maintains the abundance of collagen type IV in the tunica media and vessel elasticity, preventing aneurysm formation [33]. Consistently, most reported cases of meningioma and intracranial aneurysm co-occurrence were in pre- and postmenopausal women [11-25].

Comorbidities and Habits

Among the reported cases, hypertension was the most common comorbidity (six of 19 cases, 31.58%) [11-25]. Hypertension is pathognomonic for intracranial vascular comorbidities [35]. Controlling blood pressure is important to decrease the risk of postoperative mortality and morbidity [35]. Moreover, blood pressure should be tightly controlled in the long term to reduce the probability of recurrence [35]. Persistently high blood pressure can easily accelerate arteriosclerosis and lead to aneurysm formation [35]. Moreover, an increase in blood pressure weakens the arterial wall, a phenomenon that can lead to rupture and SAH [35,36]. Atherosclerosis and diabetes mellitus are two faces of the same coin: both can exert a stenotic pathological effect that leads to the pathophysiology of an aneurysm [37]. Maintaining glycated hemoglobin and the lipid profile in the normal range can be especially helpful in preventing aneurysm formation [37]. However, a study showed that diabetes does not exert a risk on the development of intracranial aneurysms [37]. Surprisingly, it can be protective against aneurysmal rupture [37]. Moreover, there is a low prevalence of SAH among patients with diabetes mellitus [37]. Nevertheless, we suggest controlling blood sugar to be on the safe side. Ulcerative colitis has a very rare extraintestinal manifestation known as leukocytoclastic vasculitis [38]. Due to its rarity, considering it as an etiological or risk factor may not be logical [38]. In addition, other autoimmune diseases related to ulcerative colitis do not lead to intracranial vascular problems [38]. Other comorbidities do not necessarily correlate with meningioma and/or aneurysms [5,6]. Although the comorbidities by themselves are a causative agent or high-risk factors for the aneurysm, meningioma adventitial adhesive pressure can participate in the process along with the other comorbidities; however, this does not mean that a meningioma always directly causes an intracranial aneurysm [5,6]. Unfortunately, as none of the included cases described the patients’ habits, we cannot determine whether smoking or alcoholism are linked to meningioma and intracranial aneurysm co-occurrence [11-25].

Symptomatology

Among all cases, headache was the most reported symptom by the patients (11 of 19 cases, 57.89%)[11-25]. Other common symptoms such as left ptosis and diplopia are highly related to the presence of a sellar meningioma. A headache itself is not particularly helpful as a prognostic tool. Although some patients have described it as the worst headache of their entire lives, it could be a sign of SAH as a vascular complication or other symptoms such as speech disturbance. This clinical neurosurgical point of view should serve as the initiator to decide whether the management should be started with tumor resection or blocking the aneurysm. The choice depends on the patient’s status. For example, if the patient presents with SAH, it would not be wise to consider tumor resection.

Meningioma Locations

The frontal and clinoid locations were the most common in the case reports (each in four of 19 cases, 21.05%) [11-25]. This finding agrees with a study of the preferred meningioma location that reported 20.8% of 1,107 cases to be convexity meningioma [3]. The location influences the feasibility of an intervention as well as the postoperative outcomes [11]. The first reported case involved a left frontal meningioma and led to an uneventful prognosis, but the patient still complained of headaches [11]. This outcome may be due to the absence of case reports and lack of experience of meningioma and intracranial aneurysm co-occurrence [14]. In another case, left anterior clinoidal meningioma resulted in mild right hemiparesis due to hemorrhage in the left Sylvian fissure [14]. Compared with other clinoid meningiomas, this patient had a poor prognosis, perhaps because the surgeon did not resect the tumor and clip the aneurysm at the same time [15]. A meningioma accompanied by a right MCA or left ACA aneurysm could lead to difficulty in controlling hemodynamics [15]. In such a case, it would be better to separate the tumor and aneurysm interventions into two operations to ensure patient stability [15]. In the case of an intrahemispheric meningioma, embolization of the distal left pericallosal artery was done to decrease the probability of rupture [15]. However, angiography revealed recurrence after three days [15]. After tumor resection, the probability of bleeding was 0.2%, especially in the first six months, and only 9.1% of the patients who had a recurrence required retreatment [39].

Meningioma Histopathology

Because the cases we found were reported from a neurosurgical point of view, the majority (10 of 19 cases, 52.63%) did not include histopathological details [11-25]. Of the remaining nine cases, eight were grade I, with the majority (five of eight cases, 62.5%) being meningothelial, the most common subtype [39]. Only one case was grade II (atypical subtype) [40]. Consistent with the epidemiological nature of grade II tumors, the patient was male [40]. The histopathological presentation could assist in treatment decisions because the lower the meningioma grade, the greater the intraoperative ability to resect the tumor [40,41]. There is also less of a chance that an aneurysm could rupture [40]. With higher meningioma grades, there is greater adventitial adherence, which requires more arterial manipulation during the operation [40].

Aneurysmal Artery

We noted a relatively equal distribution of ICA, ACA, MCA, AcomA, and PcomA aneurysms [42]. These arteries are highly perfused or part of the intercommunicating areas and are more susceptible to an aneurysm [42]. The most frequent type of ICA is an AcomA aneurysm, which is more frequently associated with meningioma and is expected to occur in 2%-5% of cases [13]. In addition, skull base tumors are more likely to develop aneurysms than convexity tumors [43]. Moreover, ICA and vertebrobasilar artery aneurysms are more commonly associated with skull base tumors, whereas MCA and ACA aneurysms are more likely to accompany convexity tumors [43]. Along with their physio-anatomical nature, a meningioma growing toward the arterial wall can lead to a catastrophic result such as rupture and bleeding [44]. Moreover, rebleeding of ruptured aneurysms occurs in approximately 65% of cases during the second instance and in 85% of cases during the third instance [44].

Imaging Investigations

The diagnosis depends much more on neuroimaging than the clinical picture with which the patient presents [24]. Symptoms that arise from brain tumors can progress gradually or rapidly [24]. On the other hand, symptoms of vascular diseases tend to be sudden [24]. Furthermore, the symptoms of a brain tumor such as a meningioma can be mimicked by other lesions [24]. Therefore, advanced investigation modalities are required to distinguish between them [24]. Unfortunately, these modalities are not available in many low- and middle-income countries [24]. Meningioma presents iso-intensity on T1-weighted magnetic resonance imaging sequences and hyperintensity on T2-weighted and fluid-attenuated inversion recovery sequences [23,43]. Moreover, the dural-tail sign can indicate the presence of meningioma [23,43]. Even though magnetic resonance angiography (MRA) has a high sensitivity to detect an intracranial aneurysm, it might be more challenging to detect smaller or occult aneurysms [23,43]. In these cases, it might be helpful to use digital subtraction angiography [23,43]. Because this modality can detect small and more occult aneurysms, it is considered the gold standard for intracranial aneurysms and is the best measure to be used postoperatively for follow-up [23,43]. Nevertheless, MRA should be performed preoperatively in patients with a brain tumor to visualize neoplastic vascularization and identify an incidental aneurysm that may lead to a disaster if left undetected [45].

Management

The management of meningiomas and aneurysms is a therapeutic challenge, and there is no consensus on the best approach [46]. Which should be treated first? Historically, this has been a neurosurgical dilemma since the coexistence of meningioma with the intracranial aneurysm was first reported in 1944 by Arieti et al. [46]. In patients with both aneurysms and meningioma, the symptomatic lesion is treated first [46]. If the patient presents with SAH, this condition must be treated before surgery to remove the tumor [46]. Meningioma and aneurysms can be managed simultaneously if they are near each other and if the surgeon can clip the aneurysm unless it is intramural [46-52]. In such a case, the aneurysm should be endovascularly coiled before tumor excision [46-52]. Moreover, incidental aneurysms are better managed conservatively [46-52]. It is crucial to promptly address ruptured aneurysms through surgical clipping or endovascular coiling to prevent rebleeding [18]. In cases where an aneurysm and meningioma are located in close proximity or adjacent to each other, it may be possible to manage them together, thus avoiding the need for additional surgical procedures and potential complications [18]. However, if an aneurysm and meningioma are on opposite sides (contralateral) and cannot be treated simultaneously, it is advisable to prioritize the healing of a ruptured aneurysm before considering a separate procedure to address an unruptured aneurysm or meningioma [18]. This approach ensures that sufficient healing can occur before undertaking additional interventions [18]. We would like to emphasize an important point: following the principle of “first, do no harm,” the treatment of an unruptured intracranial aneurysm may not be necessary [53]. According to a prospective study, the three- and five-year rupture rates of unruptured aneurysms in the same region were as low as 0.6% and 0.4%, respectively [53].

## Conclusions

Based on the case reports in the literature, the co-occurrence of meningioma and aneurysm can usually be treated effectively, with postoperative symptom resolution and no recurrence. Preoperative investigational imaging can dramatically affect the final outcome. Simultaneous management can be used in most cases unless the patient has a higher meningioma grade, multiple aneurysms, or a ruptured aneurysm (in which case the aneurysm should be treated first). Because cases of meningioma and aneurysm co-occurrence are rare, we encourage every neurosurgical department to share and report such cases.

## Appendices

**Table 3:** CASP systematic review checklist.

Study	Q1	Q2	Q3	Q4	Q5	Q8	Q9	Q10
Javalkar et al., 2009 [11]	Yes	Yes	Yes	Yes	Yes	Yes	Yes	Yes
Shigemori et al., 1991 [13]	Yes	Yes	Yes	Yes	Yes	Yes	Yes	Yes
Suslu et al., 2009 [14]	Yes	Yes	Yes	Yes	Yes	Yes	Yes	Yes
Paraskevopoulos et al., 2011 [15]	Yes	Yes	Yes	Yes	Yes	Yes	Yes	Yes
Alnaami et al., 2013 [16]	Yes	Yes	Yes	Yes	Yes	Yes	Yes	Yes
Waqas et al., 2015 [17]	Yes	Yes	Yes	Yes	Yes	Yes	Yes	Yes
Eulate-Beramendi et al., 2017 [18]	Yes	Yes	Yes	Yes	Yes	Yes	Yes	Yes
Zhou et al., 2017 [19]	Yes	Yes	Yes	Yes	Yes	Yes	Yes	Yes
Yip et al., 2019 [20]	Yes	Yes	Yes	Yes	Yes	Yes	Yes	Yes
Papadimitriou et al., 2020 [21]	Yes	Yes	Yes	Yes	Yes	Yes	Yes	Yes
Tanaka et al., 2022 [22]	Yes	Yes	Yes	Yes	Yes	Yes	Yes	Yes
Algburi et al., 2022 [23]	Yes	Yes	Yes	Yes	Yes	Yes	Yes	Yes
Wei et al., 2022 [24]	Yes	Yes	Yes	Yes	Yes	Yes	Yes	Yes
Onyia et al., 2023 [25]	Yes	Yes	Yes	Yes	Yes	Yes	Yes	Yes

**Table 4:** CASP systematic review checklist.

Study	Q6	Q7
Javalkar et al., 2009 [11]	This study included 5 case reports, in which all patients were females. There was diversity in the affected arteries. All of them underwent complete tumor resection/aneurysmal clipping. All patients had full recovery after the surgery	Since it is a series of case reports, no confidence interval was mentioned to measure the accuracy of the data
Shigemori et al., 1991 [13]	The study recorded the first case report of this phenomenon highlighting how the neurosurgical field improved over time. Uneventful prognosis. This may be due to the specific (MCA artery) involvement	Since it is a case report, no confidence interval was mentioned to measure the accuracy of the data
Suslu et al., 2009 [14]	It is a case of left frontal meningioma accompanied by an aneurysm of the cavernous segment of ICA. Only tumor resection was done with expectant management for the aneurysm because ICA is a dangerous artery to operate and symptoms were not related to the aneurysm. The patient fully recovered after the surgery	Since it is a case report, no confidence interval was mentioned to measure the accuracy of the data
Paraskevopoulos et al., 2011 [15]	It is a case of left anterior clinoidal meningioma accompanied by right MCA and left ACA. They took the risk to clip these two big arteries along with complete resection. This resulted in hemorrhage in the left Sylvian fissure/mild right hemiparesis	Since it is a case report, no confidence interval was mentioned to measure the accuracy of the data
Alnaami et al., 2013 [16]	It is a case of intrahemispheric meningioma accompanied by the distal left pericallosal artery aneurysm. The application of endovascular embolization played a significant role in the prognosis of the case. The patient fully recovered after the surgery	Since it is a case report, no confidence interval was mentioned to measure the accuracy of the data
Waqas et al., 2015 [17]	It is a case of left clinoidal meningioma accompanied by a left ICA aneurysm. The patient underwent complete tumor resection/aneurysmal clipping. The patient fully recovered after the surgery	Since it is a case report, no confidence interval was mentioned to measure the accuracy of the data
Eulate-Beramendi et al., 2017 [18]	Two cases of left sphenoidal and posterior meningioma, both PcomA, were affected. Low preoperative measures such as the presence of incidental aneurysms showed how much it could affect the future prognosis	Since it is a case report, no confidence interval was mentioned to measure the accuracy of the data
Zhou et al., 2017 [19]	It is a case of left medial sphenoidal wing meningioma. It is the first case report that involved an extensive ICA aneurysm reaching up to its ophthalmic branch. A good decision was made to embolize the aneurysm due to its challenging location and over manipulation like surgical clipping was deferred. The procedure saved the patient’s life	Since it is a case report, no confidence interval was mentioned to measure the accuracy of the data
Yip et al., 2019 [20]	It is a case of right frontotemporal meningioma accompanied by an aneurysm of the bifurcation of MCA. It has a unique histopathology, in which it was found to be grade II. Specifically, it was a fibrous tumor/hemangiopericytoma. Complete tumor resection/and aneurysmal clipping were done, leading to by full recovery after the surgery	Since it is a case report, no confidence interval was mentioned to measure the accuracy of the data
Papadimitriou et al., 2020 [21]	It is a case of falcine meningioma. Three aneurysms were found, namely, the left pericallosal artery, left AcomA, and right frontopolar artery. Endovascular embolization for the aneurysms before tumor resection was applied due to the high risk of unexpected hemodynamic dysregulation during surgery The patient had a full recovery after the surgery due to wise decision-making	Since it is a case report, no confidence interval was mentioned to measure the accuracy of the data
Tanaka et al., 2022 [22]	It is a case of right frontal meningioma. The aneurysm in this case was the left ICA-OpthA artery. The aneurysm was embolized and then the tumor was fully resected. The patient had a full recovery after the surgery	Since it is a case report, no confidence interval was mentioned to measure the accuracy of the data
Algburi et al., 2022 [23]	It is a case of a right frontal meningioma accompanied by an ipsilateral AcomA aneurysm. Preoperative measures such as diagnostic catheter angiography revealed the capability of complete tumor resection along with aneurysmal clipping. The patient had a full recovery after the surgery	Since it is a case report, no confidence interval was mentioned to measure the accuracy of the data
Wei et al., 2022 [24]	It is a case of a right paraflacine meningioma accompanied by AcomA and A1, A2 of right ACA. Even though the location of this meningioma and the affected arteries were hard to manage, radiological measures indicated the capability of the simultaneous surgical intervention of tumor resection and aneurysmal clipping. The patient had a full recovery after the surgery	Since it is a case report, no confidence interval was mentioned to measure the accuracy of the data
Onyia et al, 2023 [25]	It is a case of sellar meningioma accompanied by left PcomA. Meningioma in this case was completely resected with preserved pituitary function. Moreover, the aneurysm was clipped successfully. The patient had a full recovery after the surgery	Since it is a case report, no confidence interval was mentioned to measure the accuracy of the data

CASP systematic review checklist/Questions

1.      Did the review address a clearly focused question?

2.      Did the authors look for the right type of papers?

3.      Do you think all the important, relevant studies were included?

4.      Did the review’s authors do enough to assess quality of the included studies?

5.      If the results of the review have been combined, was it reasonable to do so?

6.      What are the overall results of the review?

7.      How precise are the results?

8.      Can the results be applied to the local population?

9.      Were all important outcomes considered?

10.    Are the benefits worth the harms and costs?

## References

[1] Wang Z, Ma J, Yue H (2023). Vascular smooth muscle cells in intracranial aneurysms. Microvasc Res.

[2] Vega C, Kwoon JV, Lavine SD (2002). Intracranial aneurysms: current evidence and clinical practice. Am Fam Physician.

[3] Xu Z, Rui YN, Hagan JP, Kim DH (2019). Intracranial aneurysms: pathology, genetics, and molecular mechanisms. Neuromolecular Med.

[4] Williams LN, Brown RD Jr (2013). Management of unruptured intracranial aneurysms. Neurol Clin Pract.

[5] Sforza DM, Putman CM, Cebral JR (2009). Hemodynamics of cerebral aneurysms. Annu Rev Fluid Mech.

[6] Alruwaili AA, De Jesus O (2023). Meningioma. https://www.ncbi.nlm.nih.gov/books/NBK560538/.

[7] Torp SH, Solheim O, Skjulsvik AJ (2022). The WHO 2021 Classification of Central Nervous System tumours: a practical update on what neurosurgeons need to know-a minireview. Acta Neurochir (Wien).

[8] Franca RA, Della Monica R, Corvino S, Chiariotti L, Del Basso De Caro M (2023). WHO grade and pathological markers of meningiomas: clinical and prognostic role. Pathol Res Pract.

[9] Handa J, Matsuda I, Handa H (1976). Association of brain tumor and intracranial aneurysms. Surg Neurol.

[10] Taylor PE (1961). Delayed postoperative hemorrhage from intracranial aneurysm after craniotomy for tumor. Neurology.

[11] Javalkar V, Guthikonda B, Vannemreddy P, Nanda A (2009). Association of meningioma and intracranial aneurysm: report of five cases and review of literature. Neurol India.

[12] (2023). Critical appraisal skills programme. http://uk.net/.

[13] Shigemori M, Tokunaga T, Miyagi J, Eguchi G, Kuramoto S, Irie K, Morimatsu M (1991). Multiple brain tumors of different cell types with an unruptured cerebral aneurysm--case report. Neurol Med Chir (Tokyo).

[14] Suslu HT, Bozbuga M (2011). Primary brain tumors associated with cerebral aneurysm: report of three cases. Turk Neurosurg.

[15] Paraskevopoulos D, Magras I, Balogiannis I, Polyzoidis K (2011). Anterior clinoidal meningioma coincidental with bilateral intracranial aneurysms. Hippokratia.

[16] Alnaami I, Ho P, Lu JQ, Wheatley B (2013). Case report: meningioma with intra-tumoural haemorrhage secondary to ruptured distal anterior cerebral artery aneurysm. Open Neuroimag J.

[17] Waqas M, Hadi YB, Ujjan B, Javed G (2015). Clinoidal meningioma associated with an internal carotid artery aneurysm. BMJ Case Rep.

[18] Eulate-Beramendi S, Alvarez-Vega MA, Gutierrez-Morales JC, Lopez-Garcia A (2017). Meningioma associated with brain aneurysm: report of two cases. Turk Neurosurg.

[19] Zhou X, Din Z, Liu H, Li Y (2017). Multiple intracranial aneurysms concurrent with a clinoid meningioma: a case report. Turk Neurosurg.

[20] Papadimitriou K, Rocca A, Dunet V, Daniel RT (2020). Feeding artery aneurysms associated with large meningiomas: case report and review of the literature. Heliyon.

[21] Yip CM, Lee HP, Fu JH, Hsu SH (2019). Coexistence of intracranial solitary fibrous tumor/hemangiopericytoma and right middle cerebral artery aneurysm. J Surg Case Rep.

[22] Tanaka S, Kobayashi M, Ichinose T (2022). Intraoperative rupture of intracerebral aneurysm immediately after meningioma resection: a case report. BMC Neurol.

[23] Algburi HA, Sharma M, Ismail M (2022). The coexistence of anterior communicating artery aneurysm and meningioma: a literature review and illustrative case. Surg Neurol Int.

[24] Wei RJ, Wu XL, Xia F, Chen JC (2022). Case report and literature review: treatment of multiple meningiomas combined with multiple unruptured aneurysms in a single operation. Front Surg.

[25] Onyia C, Ojo O, Arekhandia B (2023). Sellar brain tumour co-existing with a left posterior communicating aneurysm causing ptosis: lessons learnt (case report). Pan Afr Med J.

[26] Texakalidis P, Sweid A, Mouchtouris N (2019). Aneurysm formation, growth, and rupture: the biology and physics of cerebral aneurysms. World Neurosurg.

[27] Pia HW, Obrador S, Martin JG (1972). Association of brain tumours and arterial intracranial aneurysms. Acta Neurochir (Wien).

[28] Kandel E, Ludkovskaya I, Dobjansky N (1986). Aneurysm inside meningioma. Case report. Acta Neurochir (Wien).

[29] Kim YH, Lee YJ, Han JH (2015). Association of intracranial aneurysms and meningiomas: a case-control study. J Neurosurg.

[30] Goodman ML, Nelson PB (1988). Association of an epidermoid tumor with an aneurysm of the anterior communicating artery. Neurosurgery.

[31] Pant B, Arita K, Kurisu K, Tominaga A, Eguchi K, Uozumi T (1997). Incidence of intracranial aneurysm associated with pituitary adenoma. Neurosurg Rev.

[32] Korhonen K, Salminen T, Raitanen J, Auvinen A, Isola J, Haapasalo H (2006). Female predominance in meningiomas can not be explained by differences in progesterone, estrogen, or androgen receptor expression. J Neurooncol.

[33] Inagawa T, Hirano A (1990). Autopsy study of unruptured incidental intracranial aneurysms. Surg Neurol.

[34] Khalil RA (2013). Estrogen, vascular estrogen receptor and hormone therapy in postmenopausal vascular disease. Biochem Pharmacol.

[35] Wajngarten M, Silva GS (2019). Hypertension and stroke: update on treatment. Eur Cardiol.

[36] Tada Y, Wada K, Shimada K (2014). Roles of hypertension in the rupture of intracranial aneurysms. Stroke.

[37] Bae HJ, Choi JH, Kim BS, Lee KS, Shin YS (2019). Predictors of atherosclerotic change in unruptured intracranial aneurysms and parent arteries during clipping. World Neurosurg.

[38] Pantic I, Jevtic D, Nordstrom CW, Madrid C, Milovanovic T, Dumic I (2022). Clinical manifestations of leukocytoclastic vasculitis, treatment, and outcome in patients with ulcerative colitis: a systematic review of the literature. J Clin Med.

[39] Crobeddu E, Lanzino G, Kallmes DF, Cloft HJ (2013). Review of 2 decades of aneurysm-recurrence literature, part 2: managing recurrence after endovascular coiling. AJNR Am J Neuroradiol.

[40] Sun C, Dou Z, Wu J (2020). The preferred locations of meningioma according to different biological characteristics based on voxel-wise analysis. Front Oncol.

[41] Poulen G, Vignes JR, Le Corre M, Loiseau H, Bauchet L (2020). WHO grade II meningioma: epidemiology, survival and contribution of postoperative radiotherapy in a multicenter cohort of 88 patients. Neurochirurgie.

[42] Keedy A (2006). An overview of intracranial aneurysms. Mcgill J Med.

[43] Petrecca K, Sirhan D (2009). Paraclinoid aneurysm concealed by sphenoid wing meningioma. Acta Neurochir (Wien).

[44] Rosenørn J, Eskesen V, Schmidt K, Rønde F (1987). The risk of rebleeding from ruptured intracranial aneurysms. J Neurosurg.

[45] Goldbrunner R, Stavrinou P, Jenkinson MD (2021). EANO guideline on the diagnosis and management of meningiomas. Neuro Oncol.

[46] Zhong Z, Sun Y, Lin D, Sun Q, Bian L (2013). Surgical treatment of brain tumor coexisted with intracranial aneurysm--case series and review of the literature. Neurosurg Rev.

[47] Takeda N, Nishihara M, Yamanishi S, Kidoguchi K, Hashimoto K (2017). Strategy for patients with co-existence of meningioma and intracerebral aneurysm, especially unruptured aneurysm (-seven cases and review of the literature-). J Clin Neurosci.

[48] Tancioni F, Egitto MG, Tartara F (1998). Aneurysm occurring within a meningioma: case report. Br J Neurosurg.

[49] Cha KY, Park SK, Hwang YS, Kim TH (2005). Strategy for the patient with tuberculum sellae meningioma combining bilateral internal artery aneurysm. J Korean Neurosurg Soc.

[50] O'Neill OR, Barnwell SL, Silver DJ (1995). Middle meningeal artery aneurysm associated with meningioma: case report. Neurosurgery.

[51] Lama M, Mottolese C (2000). Middle meningeal artery aneurysm associated with meningioma. J Neurosurg Sci.

[52] Javadpour M, Khan AD, Jenkinson MD, Foy PM, Nahser HC (2004). Cerebral aneurysm associated with an intracranial tumour: staged endovascular and surgical treatment in two cases. Br J Neurosurg.

[53] Tominari S, Morita A, Ishibashi T (2015). Prediction model for 3-year rupture risk of unruptured cerebral aneurysms in Japanese patients. Ann Neurol.

